# Supplementary material for: Evaluating task shifting to the clinical technologist in Dutch healthcare: A mixed methods study
Source: PLoS One. 2023 Mar 1;18(3):e0281053. doi: 10.1371/journal.pone.0281053 (PMC9976997; doi:10.1371/journal.pone.0281053)
Supplement: Attachment — Submitted filename: Response to reviewers (08.10.2022).docx [file pone.0281053.s001.docx]

*Dear editorial team,*

*A point-by-point response to the comments made the editorial team and reviewers is provided below. For readability we have written our comments in italics and coloured them red.*

*Kind regards,*

*also on behalf of the other authors,*

*Maarten de Haan (corresponding author)*

PONE-D-21-40694

The Effects of Task Shifting to a New Profession in Dutch Healthcare  - A Mixed Methods Study Involving the Clinical Technologist

PLOS ONE

Dear Dr. de Haan,

Thank you for submitting your manuscript to PLOS ONE. After careful consideration, we feel that it has merit but does not fully meet PLOS ONE’s publication criteria as it currently stands. Therefore, we invite you to submit a revised version of the manuscript that addresses the points raised during the review process.

Please submit your revised manuscript by May 23 2022 11:59PM. If you will need more time than this to complete your revisions, please reply to this message or contact the journal office at [plosone@plos.org](mailto:plosone@plos.org). When you're ready to submit your revision, log on to <https://www.editorialmanager.com/pone/> and select the 'Submissions Needing Revision' folder to locate your manuscript file.

Please include the following items when submitting your revised manuscript:

- A rebuttal letter that responds to each point raised by the academic editor and reviewer(s). You should upload this letter as a separate file labeled 'Response to Reviewers'.
- A marked-up copy of your manuscript that highlights changes made to the original version. You should upload this as a separate file labeled 'Revised Manuscript with Track Changes'.
- An unmarked version of your revised paper without tracked changes. You should upload this as a separate file labeled 'Manuscript'.

If you would like to make changes to your financial disclosure, please include your updated statement in your cover letter. Guidelines for resubmitting your figure files are available below the reviewer comments at the end of this letter.

If applicable, we recommend that you deposit your laboratory protocols in protocols.io to enhance the reproducibility of your results. Protocols.io assigns your protocol its own identifier (DOI) so that it can be cited independently in the future. For instructions see: [https://journals.plos.org/plosone/s/submission-guidelines#loc-laboratory-protocols](https://eur01.safelinks.protection.outlook.com/?url=https%3A%2F%2Fjournals.plos.org%2Fplosone%2Fs%2Fsubmission-guidelines%23loc-laboratory-protocols&data=04%7C01%7Cmah.de.haan%40mumc.nl%7Ceecdeeb4d9574eb7376b08da196d853c%7C08273b66221040cd8b205cf6cddc279a%7C0%7C1%7C637850255424185395%7CUnknown%7CTWFpbGZsb3d8eyJWIjoiMC4wLjAwMDAiLCJQIjoiV2luMzIiLCJBTiI6Ik1haWwiLCJXVCI6Mn0%3D%7C3000&sdata=R7yg9smX9shTomlQz2C3OtJ3SSCzI5bGk0q5ceNXmu8%3D&reserved=0). Additionally, PLOS ONE offers an option for publishing peer-reviewed Lab Protocol articles, which describe protocols hosted on protocols.io. Read more information on sharing protocols at [https://plos.org/protocols?utm_medium=editorial-email&utm_source=authorletters&utm_campaign=protocols](https://eur01.safelinks.protection.outlook.com/?url=https%3A%2F%2Fplos.org%2Fprotocols%3Futm_medium%3Deditorial-email%26utm_source%3Dauthorletters%26utm_campaign%3Dprotocols&data=04%7C01%7Cmah.de.haan%40mumc.nl%7Ceecdeeb4d9574eb7376b08da196d853c%7C08273b66221040cd8b205cf6cddc279a%7C0%7C1%7C637850255424185395%7CUnknown%7CTWFpbGZsb3d8eyJWIjoiMC4wLjAwMDAiLCJQIjoiV2luMzIiLCJBTiI6Ik1haWwiLCJXVCI6Mn0%3D%7C3000&sdata=GCEoVt2VrpGzJUd5p%2Fatm5kUDG0gU%2BS32QV%2Fzkg%2Fx0c%3D&reserved=0).

We look forward to receiving your revised manuscript.

Kind regards,

Conor Gilligan
Academic Editor
PLOS ONE

Journal Requirements:

When submitting your revision, we need you to address these additional requirements.

1. Please ensure that your manuscript meets PLOS ONE's style requirements, including those for file naming. The PLOS ONE style templates can be found at

[https://journals.plos.org/plosone/s/file?id=wjVg/PLOSOne_formatting_sample_main_body.pdf](https://eur01.safelinks.protection.outlook.com/?url=https%3A%2F%2Fjournals.plos.org%2Fplosone%2Fs%2Ffile%3Fid%3DwjVg%2FPLOSOne_formatting_sample_main_body.pdf&data=04%7C01%7Cmah.de.haan%40mumc.nl%7Ceecdeeb4d9574eb7376b08da196d853c%7C08273b66221040cd8b205cf6cddc279a%7C0%7C1%7C637850255424185395%7CUnknown%7CTWFpbGZsb3d8eyJWIjoiMC4wLjAwMDAiLCJQIjoiV2luMzIiLCJBTiI6Ik1haWwiLCJXVCI6Mn0%3D%7C3000&sdata=lCnmpHT3Mj%2B202SdlARds0ezBYQzIRZrFpGJ4BlAdEE%3D&reserved=0)  and

[https://journals.plos.org/plosone/s/file?id=ba62/PLOSOne_formatting_sample_title_authors_affiliations.pdf](https://eur01.safelinks.protection.outlook.com/?url=https%3A%2F%2Fjournals.plos.org%2Fplosone%2Fs%2Ffile%3Fid%3Dba62%2FPLOSOne_formatting_sample_title_authors_affiliations.pdf&data=04%7C01%7Cmah.de.haan%40mumc.nl%7Ceecdeeb4d9574eb7376b08da196d853c%7C08273b66221040cd8b205cf6cddc279a%7C0%7C1%7C637850255424185395%7CUnknown%7CTWFpbGZsb3d8eyJWIjoiMC4wLjAwMDAiLCJQIjoiV2luMzIiLCJBTiI6Ik1haWwiLCJXVCI6Mn0%3D%7C3000&sdata=KgHMvJfHqaDRqx79CC3%2FXT%2F1T1Bi5dByuaSEVPn%2B2FA%3D&reserved=0)

2. Please include your full ethics statement in the ‘Methods’ section of your manuscript file. In your statement, please include the full name of the IRB or ethics committee who approved or waived your study, as well as whether or not you obtained informed written or verbal consent. If consent was waived for your study, please include this information in your statement as well.

*Response from authors:*

*We have included an ethics statement and inserted a reference to the ethics committee decision regarding this particular study.*

3. In your Data Availability statement, you have not specified where the minimal data set underlying the results described in your manuscript can be found. PLOS defines a study's minimal data set as the underlying data used to reach the conclusions drawn in the manuscript and any additional data required to replicate the reported study findings in their entirety. All PLOS journals require that the minimal data set be made fully available. For more information about our data policy, please see <http://journals.plos.org/plosone/s/data-availability>.

Upon re-submitting your revised manuscript, please upload your study’s minimal underlying data set as either Supporting Information files or to a stable, public repository and include the relevant URLs, DOIs, or accession numbers within your revised cover letter. For a list of acceptable repositories, please see <http://journals.plos.org/plosone/s/data-availability#loc-recommended-repositories>. Any potentially identifying patient information must be fully anonymized.

Important: If there are ethical or legal restrictions to sharing your data publicly, please explain these restrictions in detail. Please see our guidelines for more information on what we consider unacceptable restrictions to publicly sharing data: <http://journals.plos.org/plosone/s/data-availability#loc-unacceptable-data-access-restrictions>. Note that it is not acceptable for the authors to be the sole named individuals responsible for ensuring data access.

We will update your Data Availability statement to reflect the information you provide in your cover letter.

*Response by authors:*

*We understand and support making data transparent and accessible. However, as stated in our Data Availability statement upon first submission, we are hesitant to do so in a public depository without restrictions.*

*This is primarily because despite our efforts to anonymize the quantitative and qualitative data-sets, the small number of participants and specifics of the respondent-group still make it possible to trace data back to individuals. For example: often there was only one or two Clinical Technologists performing certain reserved procedures or working with a particular technology. Considering we promised our respondents full anonymity upon participation in our study and the fact respondents report on sensitive issues (e.g. relationship with bosses, salary, etc) we feel uncomfortable sharing the underlying data-set without cause.*

*Consequently we propose to appoint an independent individual(s) to review potential request for access to the underlying data-set. Potential individual(s) which could serve in such a role are Dr. Emmy Derks (she served as independent confident for patients throughout our study) or Marleen Groenier (she is reviewer #2 for this submission and as a previous staff member of the study for Clinical Technology at University Twente knows the particulars of our study and the sensitivities of our respondent groups).*

*We hope such a construct is sufficient to ensure Data Availability as well as ensure privacy of our respondents.*

4. We note that you have stated that you will provide repository information for your data at acceptance. Should your manuscript be accepted for publication, we will hold it until you provide the relevant accession numbers or DOIs necessary to access your data. If you wish to make changes to your Data Availability statement, please describe these changes in your cover letter and we will update your Data Availability statement to reflect the information you provide.

*Response by authors:*

*Please see our response to question 3.*

Additional Editor Comments (if provided):

The reviewers have provided some very useful feedback and suggestions on the manuscript. Please give careful consideration to the comments and consider making changes to your paper to address this.


[Note: HTML markup is below. Please do not edit.]

Reviewers' comments:

Reviewer's Responses to Questions

**Comments to the Author**

1. Is the manuscript technically sound, and do the data support the conclusions?

The manuscript must describe a technically sound piece of scientific research with data that supports the conclusions. Experiments must have been conducted rigorously, with appropriate controls, replication, and sample sizes. The conclusions must be drawn appropriately based on the data presented.

Reviewer #1: Partly

Reviewer #2: Yes

2. Has the statistical analysis been performed appropriately and rigorously?

Reviewer #1: Yes

Reviewer #2: Yes

3. Have the authors made all data underlying the findings in their manuscript fully available?

The [PLOS Data policy](https://eur01.safelinks.protection.outlook.com/?url=http%3A%2F%2Fwww.plosone.org%2Fstatic%2Fpolicies.action%23sharing&data=04%7C01%7Cmah.de.haan%40mumc.nl%7Ceecdeeb4d9574eb7376b08da196d853c%7C08273b66221040cd8b205cf6cddc279a%7C0%7C1%7C637850255424185395%7CUnknown%7CTWFpbGZsb3d8eyJWIjoiMC4wLjAwMDAiLCJQIjoiV2luMzIiLCJBTiI6Ik1haWwiLCJXVCI6Mn0%3D%7C3000&sdata=xORZVoBY4bzuFmWS%2BcxWsXa4mMt6FnW3gK9OkEYOPmQ%3D&reserved=0) requires authors to make all data underlying the findings described in their manuscript fully available without restriction, with rare exception (please refer to the Data Availability Statement in the manuscript PDF file). The data should be provided as part of the manuscript or its supporting information, or deposited to a public repository. For example, in addition to summary statistics, the data points behind means, medians and variance measures should be available. If there are restrictions on publicly sharing data—e.g. participant privacy or use of data from a third party—those must be specified.

Reviewer #1: Yes

Reviewer #2: Yes

4. Is the manuscript presented in an intelligible fashion and written in standard English?

PLOS ONE does not copyedit accepted manuscripts, so the language in submitted articles must be clear, correct, and unambiguous. Any typographical or grammatical errors should be corrected at revision, so please note any specific errors here.

Reviewer #1: Yes

Reviewer #2: Yes

5. Review Comments to the Author

Please use the space provided to explain your answers to the questions above. You may also include additional comments for the author, including concerns about dual publication, research ethics, or publication ethics. (Please upload your review as an attachment if it exceeds 20,000 characters)

Reviewer #1: The study is interesting and of relevance in its field. However, it faces several challenges (mostly related to sample size) which make it difficult if not impossible to sufficiently answer the question on effectiveness and efficiency of CT. Using the QUAL element provides additional information which is good, but the QUAN element really faces major limitations. One way would be to state from the beginning that this is an exploratory study, move away from stating that the study analyses "effectiveness and efficiency" (perhaps towards "frequency in performance of reserved medical procedures, or similar) which it cannot do statistically and tone down the conclusions in the discussion section.

*Response from authors:*

*The terms ‘effectiveness’ and ‘efficiency’ were chosen by the commissioned party (the Ministry of Health, Wellbeing and Sports). We can therefore understand why reviewer 1 has made this comment. Nevertheless, we wish to point out that our study did not evaluate the ‘effectiveness’ and ‘efficiency’ of CTs, but rather evaluated whether a legal amendment providing an Expanded Scope of Practice (ESP) to CTs was effective and effienct. The manuscript has been altered in numerous areas to ensure this miscommunication is avoided. Also, we reflect on the terms ‘effectiveness and effiency’ in our discussion.*

Please find more detailed comments below:

Introduction

- The education of the Clinical Technologist (CT) is briefly described, which is good but this is insufficient in the level of detail: there is no mention if and how many practical trainings the CTs have as part of the studies, what courses which prepare them for the skills needed to undertake reserved procedures they have to undertake, etc.

*Response by authors:*

*We we agree with the reviewer that this is important (background) information. Consequently we have elaborated this section of the introduction, and provided additional references in which the curriculum and positioning of the CT is described in more detail.*

o Later in the manuscript, it is mentioned that CTs were listed as PhD students/researchers and were doing research. Is this part of the CT education and if yes, this should be better explained in the introduction. As this is a new role, it should be better described

*Response by authors:*

*A PhD is not part of the curriculum to become a Clinical Technologist. In order to avoid confusion on this issue, we have added “CT Graduates” in the paragraph in which PhD positions were mentioned.*

- “Studies evaluating task shifting to established professionals groups, like Nurse Practitioners (NP’s) and Physician Assistants (PA’s) have highlighted that it improves the quality of care, increases patient satisfaction, reduces workforce shortages as well as increases the overall effectiveness and efficiency of care (2, 4-9)”: This sentence is too generic and does not fully capture the evidence base on the effects of NPs and PAs internationally. The evidence is not as straightforward as it suggests in the sentence.

*Response by authors:*

*We have adjusted this sentence to reflect the concerns of the reviewer, by stating task shifting can have this effect in certain situations.*

Methods
- The methods section faces several limitations: reporting should be improved: e.g. sample size calculation, recruitment, quality assurance during data collection, planned testing of the hypotheses? What were the hypotheses?
- Ethics: the name of the ethics committee was left blank as was the reference number? “The Ethics committee of the [NAME INSTITUTION] approved the study in August 2015 [REFERENCE NUMBER ETHICS COMMITTEE]. T”
- In red colour throughout the manuscript are several sources named “anonymous…”, It is unclear why in red and second, why there are so many sources with anonymous authorship.

*Response authors:*

*The study meets the criteria of the Mixed Methods Approsail Tool (MMAT) Hong QN, Pluye P, Fàbregues S, Bartlett G, Boardman F, Cargo M, Dagenais P, Gagnon M-P, Griffiths F, Nicolau B, O’Cathain A, Rousseau M-C, Vedel I. Mixed Methods Appraisal Tool (MMAT), version 2018. Registration of Copyright (#1148552), Canadian Intellectual Property Office, Industry Canada. The manuscript reports our study design and rationale for conducting a Mixed Methods Study. Furthermore, the quality of the Quantitative and Qualitative data have been individually appraised to ensure no important threats to trustworthiness are present Finally, we integrate our Quantitative and Qualitative data to collectively answer the research question(s) and we describe and explain divergences and inconsistencies between the two data forms.*

*We have added a paragraph related to our sampling and recruitment approach within the Methods section of the manuscript. The hypotheses of the study are decribed in the Introduction and are referred to in the Discussion sections. The name of our institution, ethics committee and references to our own work were removed to facilitate a blind review process. We have now included these elements in the revised manuscript.*

Results:
- 13 patients? This raises major concerns in terms of generalizability (not even talking about representativeness), actually, the authors should consider omitting the findings from these 13 patients
- Sample size of the CTs is also low when it comes to the analysis of the reserved procedures. This is a major limitation. In fact, the paper should move away from stating that it analyses the “effectiveness” which implies quality of care elements as well, toward e.g. the performance and frequency of performance of a set of reserved procedures.

*Response by authors:*

*We understand the concerns of the reviewer in relation to the low patient response, however, in contrast to a sequential mixed methods design, our study design does not strive for ‘generalizability of data’ but rather focuses on ‘trustworthiness of data’. The low response is referred to multiple times throughout the manuscript, and its potential consequences to our overall findings are extensively described in the Discussion section.*

*Nevertheless, we agree with the reviewer that we should be careful in our wording. Also in relation to a similar point made by the reviewers we have made changes in the introduction the manuscript to state that the study did not seek to investigate the ‘effectiveness and efficiency of CTs’ but rather explores whether the legal amendment (providing CTs a temporary ESP) is effective and efficient. Upon the suggestion of the reviewer we have also added that in this context we mean that for the legal amendment to be ‘effective’ it should be used by CTs to perform reserved procedures independently, and to be ‘efficient’ this should lead to these procedures taking up less time than when those procedures are first delegated to CTs by an MD or another professional with an independent authority to perform reserved procedures.*

Discussion
- This sentence has major issues: “While a lower QUAN response limited our ability to statistically test this hypothesis, the combined results of this mixed methods study highlight that CTs use their ESP frequently to perform five categories of reserved procedures independently (i.e., catheterizations, surgical procedures, procedures with radiation, injections and punctures). As a result, the ESP seems effective for these five reserved procedures.” - it is a limitation that the sample size was not reached and should be stated as such instead of saying that together with the QUAL element, the 5 procedures seemed to be effective.

*Response by authors:*

*The premise of mixed methods research is that the use of quantitative and qualitative data in combination provides a better understanding than either approach alone (Creswell & Plano Clark 2007). Our study is a prime example of that. While the descriptive statistics presented in our manuscript suggest the ESP is effective and efficient, the response rate was too low to statistically test these findings. However, the qualitative results corroborated these findings, providing a high level of confidence that the ESP is indeed effective and efficient for these five procedures. We agree with the reviewers that the low quantitative response rate is a limitation of our study, and we say so in our discussion section. Due to our mentioning this in the discussion, we do not feel it is necessary to alter this specific sentence.*

Reviewer #2: In this manuscript, the authors evaluate how shifting reserved procedures from physicians to an emerging profession, in this case clinical technologists (CTs), affects the effectiveness and efficiency of patient care. The authors use the conceptual framework of Donabedian to guide their analysis of the data gathered. Both quantitative and qualitative data were gathered resulting in multiple perspectives on the effectiveness and efficiency of task shifting.

The methods are clearly and elaborately described. The results are presented in a structured manner, following the conceptual framework used, and tables and figures are used adequately to support the interpretation of the data, although figures 2 and 3 are somewhat redundant given the elaborate presentation of findings in the main text. The conclusions are supported by the results.

*Response by authors:*

*The data presented in figures 2 and 3 indeed partly overlap with the presentation of the finding in the main text. While an all-text presentation is an option, we feel this would be sub-optimal. Providing a visual tool in addition to a written presentation, helps the readers quickly identify the trends we wish to highlight. Alternatively, an all-text presentation would require a substantial addition to the word-count.*

\My main concern is the readability of the manuscript. The design of the study is quite complex and many data sources are used, both quantitative and qualitative. Data from different sources are reported separately and in combination. All this makes it hard to follow which results are relevant and how they contribute to answering the overall research question. Although tables 1 and 6 are added to provide an overview of the data sources and a summary of the results, the main text has so many details that it is hard to keep track of the main outcomes of this study. This could be improved.

*Response by authors:*

*We understand the results can be hard to follow due to the sheer amount of data collected, analyzed and reported. To overcome this, the paragraph explaining how the results are reported has been moved from the methods section to the beginning of the results section. We feel that the chosen approach (to report the results using our conceptual framework and to first describe QUAL results for each part) is the most efficient way to report our findings.*

On page 4 the research question is introduced which focuses on evaluating effectiveness and efficiency. However, in the introduction, these terms are not conceptualized. From the hypothesis, it becomes clear that effectiveness is associated with an increase in reserved procedures performed by CTs and efficiency with duration but the definitions are not introduced or explained. I would encourage the authors to not only explain task shifting in the introduction but also the definition and measurement of effectiveness and efficiency in this particular context.

*Response by authors:*

*We have added a specific explaination in the introduction of how the terms ‘effectiveness’ and ‘efficiency’ were used in the context of our study. And we also reflect on these terms in the discussion section of the manuscript.*

In the conclusions on pages 26 and 27, there is a strong focus on the recommendation that is made to the Dutch Ministry of HWS. However, I find the results in light of 1) horizontal task shifting and 2) the task shifting to an emerging profession much more interesting (see page 27). For example, the authors state that ‘when pursuing task shifting to emerging professional groups, policymakers must be aware of such differences and judge accordingly’. However, it is unclear to me what the authors mean by that. How can one judge accordingly, given the results of the current study? Also, to what extent are efficiency and effectiveness adequate indicators of successful task shifting from physicians to CTs? I would like to read about the authors’ perspective on that in the discussion.

*Response by authors:*

*Considering the study was commissioned by the Dutch Ministry of Health and aimed to help the Ministry’s decision-making process on whether or not to make the ESP permanent, the results of the study were originally meant as policy recommendations. However, within a broader scientific and policy discussion we understand the comments of the reviewer. Subsequently, within the discussion section we have added what contributions this study makes to the debate surrounding ‘horizontal taskshifting’ and ‘emerging professional groups’. We have also elaborated on what we mean by “policymakers must be more aware” and provide several examples of how they can do so.*

6. PLOS authors have the option to publish the peer review history of their article ([what does this mean?](https://eur01.safelinks.protection.outlook.com/?url=https%3A%2F%2Fjournals.plos.org%2Fplosone%2Fs%2Feditorial-and-peer-review-process%23loc-peer-review-history&data=04%7C01%7Cmah.de.haan%40mumc.nl%7Ceecdeeb4d9574eb7376b08da196d853c%7C08273b66221040cd8b205cf6cddc279a%7C0%7C1%7C637850255424185395%7CUnknown%7CTWFpbGZsb3d8eyJWIjoiMC4wLjAwMDAiLCJQIjoiV2luMzIiLCJBTiI6Ik1haWwiLCJXVCI6Mn0%3D%7C3000&sdata=FUBEXFKFhDpVQCUpK4I9UuoirtDLxPW7lJfG0JkqAm4%3D&reserved=0)). If published, this will include your full peer review and any attached files.


If you choose “no”, your identity will remain anonymous but your review may still be made public.


**Do you want your identity to be public for this peer review?** For information about this choice, including consent withdrawal, please see our [Privacy Policy](https://eur01.safelinks.protection.outlook.com/?url=https%3A%2F%2Fwww.plos.org%2Fprivacy-policy&data=04%7C01%7Cmah.de.haan%40mumc.nl%7Ceecdeeb4d9574eb7376b08da196d853c%7C08273b66221040cd8b205cf6cddc279a%7C0%7C1%7C637850255424185395%7CUnknown%7CTWFpbGZsb3d8eyJWIjoiMC4wLjAwMDAiLCJQIjoiV2luMzIiLCJBTiI6Ik1haWwiLCJXVCI6Mn0%3D%7C3000&sdata=Jj3CkV11tUuHmTO0cAIN%2FsxmCwkTTCjTyehE9Mq9q9g%3D&reserved=0).

Reviewer #1: No

Reviewer #2: **Yes:** Marleen Groenier

[NOTE: If reviewer comments were submitted as an attachment file, they will be attached to this email and accessible via the submission site. Please log into your account, locate the manuscript record, and check for the action link "View Attachments". If this link does not appear, there are no attachment files.]

While revising your submission, please upload your figure files to the Preflight Analysis and Conversion Engine (PACE) digital diagnostic tool, [https://pacev2.apexcovantage.com/](https://eur01.safelinks.protection.outlook.com/?url=https%3A%2F%2Fpacev2.apexcovantage.com%2F&data=04%7C01%7Cmah.de.haan%40mumc.nl%7Ceecdeeb4d9574eb7376b08da196d853c%7C08273b66221040cd8b205cf6cddc279a%7C0%7C1%7C637850255424185395%7CUnknown%7CTWFpbGZsb3d8eyJWIjoiMC4wLjAwMDAiLCJQIjoiV2luMzIiLCJBTiI6Ik1haWwiLCJXVCI6Mn0%3D%7C3000&sdata=glIS46zYvhFkhbQfa7imVGjr0cDiVWzNhJ4AGgeBMwo%3D&reserved=0). PACE helps ensure that figures meet PLOS requirements. To use PACE, you must first register as a user. Registration is free. Then, login and navigate to the UPLOAD tab, where you will find detailed instructions on how to use the tool. If you encounter any issues or have any questions when using PACE, please email PLOS at [figures@plos.org](mailto:figures@plos.org). Please note that Supporting Information files do not need this step.

*In compliance with data protection regulations, you may request that we remove your personal registration details at any time.* [*(Remove my information/details)*](https://eur01.safelinks.protection.outlook.com/?url=https%3A%2F%2Fwww.editorialmanager.com%2Fpone%2Flogin.asp%3Fa%3Dr&data=04%7C01%7Cmah.de.haan%40mumc.nl%7Ceecdeeb4d9574eb7376b08da196d853c%7C08273b66221040cd8b205cf6cddc279a%7C0%7C1%7C637850255424185395%7CUnknown%7CTWFpbGZsb3d8eyJWIjoiMC4wLjAwMDAiLCJQIjoiV2luMzIiLCJBTiI6Ik1haWwiLCJXVCI6Mn0%3D%7C3000&sdata=5A%2BRjxKfuSRJwkdKSnRnfxPjt41W%2Fk2gvW2%2BNxUiy%2FY%3D&reserved=0)*. Please contact the publication office if you have any questions.*
