# Supplementary material for: Evaluating task shifting to the clinical technologist in Dutch healthcare: A mixed methods study
Source: PLoS One. 2023 Mar 1;18(3):e0281053. doi: 10.1371/journal.pone.0281053 (PMC9976997; doi:10.1371/journal.pone.0281053)
Supplement: Attachment — Submitted filename: Response to reviewers (11.12.2022).docx [file pone.0281053.s002.docx]

*Dear editorial team,*

*A point-by-point response to the comments made by the editorial team and reviewers is provided below. For readability we have written our comments in italics and coloured them red.*

*Kind regards,*

*also on behalf of the other authors,*

*Maarten de Haan (corresponding author)*

PONE-D-21-40694R1

Evaluating Task Shifting to the Clinical Technologist in Dutch Healthcare: A Mixed Methods Study

PLOS ONE

Dear Dr. de Haan,

Thank you for submitting your manuscript to PLOS ONE. After careful consideration, we feel that it has merit but does not fully meet PLOS ONE’s publication criteria as it currently stands. Therefore, we invite you to submit a revised version of the manuscript that addresses the points raised during the review process.

Please submit your revised manuscript by Jan 02 2023 11:59PM. If you will need more time than this to complete your revisions, please reply to this message or contact the journal office at [plosone@plos.org](mailto:plosone@plos.org). When you're ready to submit your revision, log on to https://www.editorialmanager.com/pone/ and select the 'Submissions Needing Revision' folder to locate your manuscript file.

Please include the following items when submitting your revised manuscript:

- A rebuttal letter that responds to each point raised by the academic editor and reviewer(s). You should upload this letter as a separate file labeled 'Response to Reviewers'.
- A marked-up copy of your manuscript that highlights changes made to the original version. You should upload this as a separate file labeled 'Revised Manuscript with Track Changes'.
- An unmarked version of your revised paper without tracked changes. You should upload this as a separate file labeled 'Manuscript'.

If you would like to make changes to your financial disclosure, please include your updated statement in your cover letter. Guidelines for resubmitting your figure files are available below the reviewer comments at the end of this letter.

If applicable, we recommend that you deposit your laboratory protocols in protocols.io to enhance the reproducibility of your results. Protocols.io assigns your protocol its own identifier (DOI) so that it can be cited independently in the future. For instructions see: <https://journals.plos.org/plosone/s/submission-guidelines#loc-laboratory-protocols>. Additionally, PLOS ONE offers an option for publishing peer-reviewed Lab Protocol articles, which describe protocols hosted on protocols.io. Read more information on sharing protocols at <https://plos.org/protocols?utm_medium=editorial-email&utm_source=authorletters&utm_campaign=protocols>.

We look forward to receiving your revised manuscript.

Kind regards,

Conor Gilligan
Academic Editor
PLOS ONE

Journal Requirements:

Please review your reference list to ensure that it is complete and correct. If you have cited papers that have been retracted, please include the rationale for doing so in the manuscript text, or remove these references and replace them with relevant current references. Any changes to the reference list should be mentioned in the rebuttal letter that accompanies your revised manuscript. If you need to cite a retracted article, indicate the article’s retracted status in the References list and also include a citation and full reference for the retraction notice.

Additional Editor Comments:

Thank you for your revision. The original reviewers and I have all now reviewed the revised version and will be happy to accept the paper with some very minor additional changes. Please note the comments made by both reviewers.

Some grammatical errors to correct include: on page 3, changing limited to 'limiting' in reference to the generalisability of findings from other studies; page 11 final paragraph, change 'date' to 'data'

*We have corrected these grammatical errors.*

One reviewer comments on an ongoing concern with the use of the term 'effectiveness'. I can see that you define effectiveness for your study purpose but perhaps you could provide some further clarification in the discussion to highlight the limitation of the measures of effectiveness used (no ,easure of procedure success or patient measures such as recovery time, errors etc).

*We have elaborated the section in the discussion where we discuss the use of the term ‘effectiveness’ in our study. In addition, as requested by the reviewer, we have gone through the manuscript to limit the amount of times we use this term. Where possible, we have provided in-text clarifications on what we mean by the term (please see our response to reviewer#1 below).*

Is the 258 CTs that participated the entire pool that was invited/eligible? Clarity around recruitment processes and rates is lacking.

*The 258 CTs were not the entire pool that was eligible but rather the total number of CTs that participated in the (quantitative) study. For clarity we added the total number of CTs eligible for all three measurement points (T1 n=215, T2 n=284, T3 n=315) as well as the total number of CTs invited for which we had contact information (T1 n=189, T2 n=271, T3 n=302).*

Please ensure that the example quotes provided match the description in the text. There are some places where several points are made in a paragraph but the quote following it does not directly relate to the last point made.

*We have changed the way we present the quotes in our manuscript to tackle this point. Rather than first discussing the QUAL results through an in-text presentation followed by all quotes, we have now opted to insert the relevant quote directly after the in-text discussion. This way there can be no misunderstanding how each specific in-text point relates to the presented quotations by respondents.*

[Note: HTML markup is below. Please do not edit.]

Reviewers' comments:

Reviewer's Responses to Questions

**Comments to the Author**

1. If the authors have adequately addressed your comments raised in a previous round of review and you feel that this manuscript is now acceptable for publication, you may indicate that here to bypass the “Comments to the Author” section, enter your conflict of interest statement in the “Confidential to Editor” section, and submit your "Accept" recommendation.

Reviewer #1: All comments have been addressed

Reviewer #2: (No Response)

2. Is the manuscript technically sound, and do the data support the conclusions?

The manuscript must describe a technically sound piece of scientific research with data that supports the conclusions. Experiments must have been conducted rigorously, with appropriate controls, replication, and sample sizes. The conclusions must be drawn appropriately based on the data presented.

Reviewer #1: Yes

Reviewer #2: Yes

3. Has the statistical analysis been performed appropriately and rigorously?

Reviewer #1: Yes

Reviewer #2: Yes

4. Have the authors made all data underlying the findings in their manuscript fully available?

The [PLOS Data policy](http://www.plosone.org/static/policies.action#sharing) requires authors to make all data underlying the findings described in their manuscript fully available without restriction, with rare exception (please refer to the Data Availability Statement in the manuscript PDF file). The data should be provided as part of the manuscript or its supporting information, or deposited to a public repository. For example, in addition to summary statistics, the data points behind means, medians and variance measures should be available. If there are restrictions on publicly sharing data—e.g. participant privacy or use of data from a third party—those must be specified.

Reviewer #1: Yes

Reviewer #2: Yes

5. Is the manuscript presented in an intelligible fashion and written in standard English?

PLOS ONE does not copyedit accepted manuscripts, so the language in submitted articles must be clear, correct, and unambiguous. Any typographical or grammatical errors should be corrected at revision, so please note any specific errors here.

Reviewer #1: Yes

Reviewer #2: Yes

6. Review Comments to the Author

Please use the space provided to explain your answers to the questions above. You may also include additional comments for the author, including concerns about dual publication, research ethics, or publication ethics. (Please upload your review as an attachment if it exceeds 20,000 characters)

Reviewer #1: The comments were addressed sufficiently and the paper has considerably improved. One issue remains: the authors still want to use the term effectiveness which is misleading in this context due to various reasons on which I elaborated earlier (e.g. sample size, study design, etc.). I would strongly recommend to replace the word with uptake or use it as rarely as possible throughout the manuscript.

*At the end of the introduction of our manuscript we define the terms ‘effectiveness’ and ‘efficiency’ and in table 1 we present how we operationalized these within the study. Our use of these terms are similar to previously published research on task shifting in the Netherlands (for example see: De Bruijn et al. 2018. National Mixed Methods Evaluation of the Effects of Removing Legal Barriers to Full Practice Authority of Dutch Nurse Practitioners and Physician Assistants. BMJ Open. Doi: 10.1136) and these terms have been used by the Dutch Ministry of Health for evaluating task shifting to five professional roles (Nurse Practitioners, Physician Assistants, Clinical Technologists, Allied Health Professional and Dental Hygienist).*

*Nevertheless, we understand that these terms might cause confusion for non-Dutch readers. Upon the suggestion of the reviewer we have limited the use of the term and where possible provided additional context in how these terms were applied within our study. Furthermore, as suggested by the editor, we elaborated on this issue further within the discussion section of the manuscript (please see our response to the editor for more information).*

Reviewer #2: The authors adequately addressed my concerns.

I urge the authors to do a final spell- and grammar check before submitting their final version. There is still quite a number of minor errors (e.g., singular/plural errors), especially in the added paragraphs in the introduction.

*A third party (native English speaker) has gone through the manuscript to assist us with final spell- and grammar checks.*

7. PLOS authors have the option to publish the peer review history of their article ([what does this mean?](https://journals.plos.org/plosone/s/editorial-and-peer-review-process#loc-peer-review-history)). If published, this will include your full peer review and any attached files.


If you choose “no”, your identity will remain anonymous but your review may still be made public.


**Do you want your identity to be public for this peer review?** For information about this choice, including consent withdrawal, please see our [Privacy Policy](https://www.plos.org/privacy-policy).

Reviewer #1: No

Reviewer #2: No

[NOTE: If reviewer comments were submitted as an attachment file, they will be attached to this email and accessible via the submission site. Please log into your account, locate the manuscript record, and check for the action link "View Attachments". If this link does not appear, there are no attachment files.]

While revising your submission, please upload your figure files to the Preflight Analysis and Conversion Engine (PACE) digital diagnostic tool, <https://pacev2.apexcovantage.com/>. PACE helps ensure that figures meet PLOS requirements. To use PACE, you must first register as a user. Registration is free. Then, login and navigate to the UPLOAD tab, where you will find detailed instructions on how to use the tool. If you encounter any issues or have any questions when using PACE, please email PLOS at [figures@plos.org](mailto:figures@plos.org). Please note that Supporting Information files do not need this step.
